# Risk factors for poor neurological outcomes after unilateral open-door laminoplasty: an analysis of the characteristics of ectopic bone

**DOI:** 10.1186/s13018-022-03074-9

**Published:** 2022-03-24

**Authors:** Zijian Hua, Jia Li, Wenshuai Li, Yu Zhang, Feng Wang, Linfeng Wang, Yong Shen

**Affiliations:** 1 Department of Orthopedic Surgery, The Third Hospital of Hebei Medical University, Shijiazhuang, 050051 People’s Republic of China; 2 The Key Laboratory of Orthopedic Biomechanics of Hebei Province, The Third Hospital of Hebei Medical University, Shijiazhuang, 050051 People’s Republic of China; 3 Department of Orthopedic Surgery, Heze Municipal Hospital, Heze, 274000 People’s Republic of China

**Keywords:** Ossification of the posterior longitudinal ligament, Neurological outcome, Cervical, Risk factors, Laminoplasty, Ectopic bone

## Abstract

**Background:**

The factors affecting neurological outcomes with unilateral open-door laminoplasty have been controversial. The purpose of this study was to evaluate the impact of the characteristics of ectopic bone on poor neurological outcomes after unilateral open-door laminoplasty.

**Methods:**

We retrospectively analyzed the clinical data of 112 patients who underwent unilateral open-door laminoplasty from September 2017 to September 2020. According to the Japanese Orthopedic Association score recovery rate after surgery (Japanese Orthopedic Association recovery rate ≥ 50% and < 50%, respectively), all patients were divided into “poor” and “good” groups. The characteristics of ectopic bone and the position relationship between the open side and ectopic bone (for lateral ossification) in the two groups were compared and analyzed. Univariate and multivariate analyses were used to determine the risk factors for poor neurological outcome.

**Results:**

We identified patients with a mean age of 58.39 years and a mean follow-up of 25.43 months. Sixty (53.6%) patients experienced recovery of poor neurological function. On univariable analysis, significant predictors of poor neurological recovery were occupation rate of spinal canal > 60% (*p* = 0.000), ossification extending to C2 (*p* = 0.006), lateral ossification (*p* = 0.032) and opening side on the ipsilateral side of the ectopic bone (*p* = 0.011). Multivariate logistic regression analysis revealed that the occupation rate of spinal canal > 60% (*P* = 0.003), ossification extending to C2 (*P* = 0.041) and opening the door on the ipsilateral side for lateral ossification (*P* = 0.013) were independent risk factors for poor prognosis of neurological function.

**Conclusions:**

An occupation ratio > 60% is the most important risk factor. Another one is ossification of the posterior longitudinal ligament extending to C2. Meanwhile, opening the door on the ipsilateral side is indeed a risk factor for lateral ossification. Better neurological function may be obtained by choosing the opposite side of the heterotopic bone as the open side. Therefore, the design of the surgical plan should comprehensively consider these factors.

## Introduction

Ossification of the posterior longitudinal ligament (OPLL) of the cervical spine is a heterotopic ossification of the cervical spinal ligament that can cause a significant amount of chronic pressure on the spinal cord and result in cervical myelopathy. For cases with severe neurological deficits, surgery is needed. Various surgical methods for cervical OPLL have been applied, including: anterior corpectomy and fusion, laminoplasty and laminectomy [1–3]. Unilateral open-door laminoplasty (UODL) has been considered to be ideally appropriate for patients with multi-level cervical degenerative diseases [4, 5], which enlarges the spinal canal to allow the spinal cord to float away from the ventral OPLL. Although laminoplasty has been reported to show satisfactory clinical outcomes and fewer complications than laminectomy [6], in a large couple of number clinical cases, we have found that the postoperative recovery of neurological function is different.

After the introduction of laminoplasty, many studies have evaluated the association between the characteristics of ossification, features of spinal cord compression and neurological outcomes. Yoon et al. summarized the factors related to the recovery of neurological function after surgery for cervical OPLL [7]. Although many studies have mentioned the association, these studies often involved only a limited number of factors, and multivariate regression analysis was rare [8]. Simultaneously, some scholars regarded that the relationship between the open side of the UODL and the pattern of ectopic bone (CT axial image) would affect the recovery of postoperative neurological function [9]. However, there is a lack of sufficient evidence to determine whether the characteristics of ectopic bone and the choice of the open side are important predictors of postoperative neurological recovery.

The various characteristics of ectopic bone and different open sides may be associated with the recovery of neurological functional in patients undergoing UODL. Therefore, the purpose of this study was to investigate the impact of the characteristics of ectopic bone on poor neurological outcomes after UODL using univariate and multivariate analyses.

## Material and methods

### Study populations

This study was approved by the Institutional Review Board of our hospital. Data were extracted from the medical records and radiological images of 112 patients with cervical myelopathy caused by cervical OPLL, who accepted cervical UODL performed by the same chief surgeon between 2017 and 2020 at our institutions. The inclusion criteria were as follows: (1) patients who were diagnosed with cervical OPLL with myelopathy, in whom CT scan showed that the ectopic bone was on the posterior longitudinal ligament, and (2) patients who were only treated with cervical UODL. The exclusion criteria were as follows: (1) patients diagnosed with radiculopathy or myelopathy without OPLL; (2) patients with cervical spinal cord injury, tumor history, neurological lesions, cervical spine surgery and postoperative infection; (3) patients who were inaccessible due to cerebral infarction, cerebral hemorrhage, severe trauma and other malignant conditions during the follow-up period; (4) patients whose 3-year follow-up observation was interrupted or whose data were missing; or (5) patients with a history of substance abuse. We investigated the clinical and OPLL-related features of all patients and evaluated the factors that might affect the postoperative neurological outcome. Each patient’s neurological outcome was evaluated using the Japanese Orthopedic Association score for cervical myelopathy, and the JOA score recovery rate was calculated using the following formula: (postoperative JOA score–preoperative JOA score)/(17 points–preoperative JOA score) × 100 (%). According to the JOA recovery rate, the patients were divided into good (JOA recovery rate ≥ 50%) and poor (JOA recovery rate < 50%) groups [10].

### Surgical techniques

The modified Hirabayashi method was used in surgery [2]. An incision was made along the midline of the posterior skin of the neck, approximately 13–15 cm in length. The paravertebral muscles on both sides were carefully dissected to expose the laminae and lateral mass, and the muscle attaching to the spinous process of C2 was preserved as much as possible. After excision of the spinous process, a high-speed drill was used to make a gutter at the junction of the lamina and facet joint. One side of the groove was completely cut off as the open side and the opposite side as the hinge side. Mini-titanium plates were used to connect the broken sides and maintain the open laminae. Depending on the length of the patient's ossified segments, the surgical segments were C3-6 or C3-7. Every case underwent dome-shaped laminectomy on the bottom of the C2 spinous process.

### Clinical and radiologic assessments

The following patient data were obtained: age, sex, body mass index (BMI), K-line (positive or negative) [11], type of OPLL (continuous, segmental, mixed and circumscribed) [12], shape of ectopic bone (hill-shaped or plateau-shaped) [13, 14], anteroposterior diameter of the ectopic bone, occupation rate of spinal canal (maximum stenosis of spinal canal), number of ossification levels, ossification extending to C2(yes or no), axial ossified pattern of the responsible ossification segment (lateral or central) [15] and position relationship between the open side and ectopic bone (for lateral OPLL) [9].

The Investigation Committee for Ossification of the Spinal Ligaments (part of the Japanese Ministry of Health, Labor and Welfare) established a commonly used classification system for OPLL. This system categorizes OPLL into 4 types: (1) continuous, a long lesion extending over several vertebral bodies; (2) segmental, one or several separate lesions behind the vertebral bodies; (3) mixed, a combination of the continuous and segmental types; and (4) circumscribed, mainly located posterior to a disc space [12]. A plateau-shaped ossification is characterized by a relatively narrow spinal canal without any localized massive ossification. In contrast, a hill-shaped OPLL appears as a massive beak-shaped ossification localized to certain levels [13]. The K-line can be described on lateral radiographs as a straight line connecting the midpoint of the C2 and C7 spinal canals [11]. Some studies have classified the type of OPLL as central or lateral based on its position on an axial CT scan. OPLL was categorized as central if the posterior prominence of the OPLL was located in the middle one-third of the spinal canal [15]. Patients with lateral cervical OPLL were divided into open-door contralateral or ipsilateral to the ectopic bone according to the position relationship between the open side and the largest ectopic bone [9]. All measurements were based on the maximum ectopic bone level.

### Statistical analysis

SPSS version 26 (IBM SPSS Statistics 26.0, IBM Corporation, Armonk, NY) was used to perform all statistical analyses. The normality of the measurement data was tested using the Shapiro–Wilk method (*P* > 0.05). All data conformed to a normal distribution, so the measurement data are described by the mean ± standard deviation (M ± SD). Comparisons between the two groups were analyzed using independent sample *t* test. The Chi-square test was used to compare groups regarding categorical variables. Multivariate correlation analysis was performed using all variables identified as significant at the *p* ≤ 0.05 level on univariable analysis to identify the risk factors associated with poor neurological outcomes.

## Results

### Univariate analysis results

Ultimately, a total of 112 patients were included in the current study (average age 59.71 ± 8.80 years old; 80 males and 32 females), while 6 patients with inadequate follow-up, 4 patients with incomplete radiographical data and 1 patient with stroke were excluded from the initial 123 patients. The average number of ossification levels was 4.57 ± 1.66 levels, and the average number of open-door segments was 4.46 ± 0.50. The average anteroposterior diameter of ossification was 7.05 ± 1.34 mm. The average BMI was 26.46 ± 3.53 kg/m^2^.

The pre- and postoperative JOA score averages were 9.86 ± 2.86 and 13.04 + 1.75, respectively. The average JOA recovery rate was 42.0% ± 26.7%. According to the JOA recovery rate, the patients were divided into two groups, and 52 (46.4%) and 60 (53.6%) patients were in the “good” and “poor” neurological outcome groups, respectively.

### *JOA recovery rate* ≥ *50% versus JOA recovery rate* < *50%*

Clinical parameters include mean age (58.62 ± 8.914 years vs. 60.67 ± 8.664 years, *P* = 0.220), gender ratio F/M (16:36 vs. 16:44, *P* = 0.632), BMI (26.40 ± 3.24 kg/m^2^ vs. 26.51 ± 3.79 kg/m^2^, *P* = 0.863), average number of open-door segments (4.38 ± 0.49 vs. 4.53 ± 0.50, *P* = 0.118), preoperative JOA score (9.54 ± 3.71 vs. 10.13 ± 1.84, *P* = 0.275), anteroposterior diameter of ossification (7.23 ± 1.65 mm vs. 6.88 ± 0.98 mm, *P* = 0.175), average number of ossification levels (4.77 ± 1.38 vs. 4.40 ± 1.87, *P* = 0.233), type of OPLL (segmental 4:48 vs. 8:52, *P* = 0.336; continuous 19:33 vs. 17:43, *P* = 0.354; mixed 15:37 vs. 19:41, *P* = 0.746; circumscribed 14:38 vs. 16:44, *P* = 0.976), shape of ectopic bone (hill-shaped/plateau-shaped, 20:32 vs. 28:32, *P* = 0.382), occupation ratio (> 60%: ≤ 60%, 24:28 vs. 48:12, *P* = 0.000), extension to C2 (yes/no 12:40 vs. 29:31, *P* = 0.006), axial pattern of the ectopic bone (central/lateral, 24:28 vs. 16:44, *P* = 0.032) and K-line (+/−, 36:16 vs. 44:16, *P* = 0.632). Of the 112 patients, 72 patients (64.29%) had lateral OPLL. Patients with lateral OPLL could be divided into the ipsilateral side door and the contralateral side door. (12:16 vs. 32:12, *P* = 0.011).

### Logistic regression analysis of “good” and “poor” groups

Table 1 shows that the differences in the occupation ratio, OPLL extending to C2 and axial ossified pattern between the “good” group and the “poor” group were statistically significant (*P* < 0.05), while the occupation ratio, OPLL extending to C2 and lateral OPLL in the “poor” group were significantly higher than those in the “good” group. No significant differences in age, sex, BMI, average number of ossification levels, type of OPLL, shape of ectopic bone or K-line state (+/−) were noted between the two groups (*P* > 0.05). In the comparison of JOA recovery rate in patients with laterally deviated OPLL, the difference in the open side was statistically significant (*P* < 0.05). Opening the door on the ipsilateral side to the ectopic bone in the “poor” group was significantly higher than those in the “good” group.

**Table 1:** Comparison of factors in the “good” and “poor” groups

Variable	JOA recovery	JOA recovery	*X*^2^/*t*	*p*
	Rate ≥ 50% (*n* = 52)	Rate < 50% (*n* = 60)		
Female Male/	16/36	16/44	*X*^2^ = 0.230	0.632
Age (years)	58.62 ± 8.91	60.67 ± 8.96	*t* = − 1.233	0.220
BMI (kg/m2)	26.40 ± 3.24	26.51 ± 3.79	*t* = − 0.173	0.863
Number of open-door segments	4.38 ± 0.49	4.53 ± 0.50	*t* = − 1.577	0.118
Preoperative JOA score	9.54 ± 3.71	10.13 ± 1.84	*t* = − 1.098	0.275
Anteroposterior diameter of OPLL (mm)	7.23 ± 1.65	6.88 ± 0.98	*t* = 1.364	0.175
No. of ossification levels	4.77 ± 1.38	4.40 ± 1.87	*t* = 1.198	0.233
*Type of OPLL*				
Segmental (Y/N)	4:48	8:52	*X*^2^ = 0.927	0.336
Continuous (Y/N)	19:33	17:43	*X*^2^ = 0.860	0.354
Mixed (Y/N)	15:37	19:41	*X*^2^ = 0.105	0.746
Circumscribed (Y/N)	14:38	16:44	*X*^2^ = 0.001	0.976
Shape of OPLL, H/P	20:32	28:32	*X*^2^ = 0.766	0.382
Occupation ratio > 60% (Y/N)	24:28	48:12	*X*^2^ = 13.90	0.000*
Axial ossified pattern, C/L	24:28	16:44	*X*^2^ = 4.608	0.032*
K-line, + / −	36:16	44:16	*X*^2^ = 0.230	0.632
Extension to C2 (Y/N)	12:40	29:31	*X*^2^ = 7.657	0.006*
Open Side (lateral OPLL), ipsi/contra	12:16	32:12	*X*^2^ = 6.424	0.011*

*There was significant differences between two groups (*P* < 0.05)

H, Hill-shaped; P, plateau-shaped; Y, yes; N, no; C, central; L, lateral; ipsi, ipsilateral side; and contra, contralateral side

Based on univariate analysis results, four indicators were used as independent variables, including the occupation ratio, OPLL extending to C2, axial ossified pattern and different sides of the door (lateral OPLL). Multivariate regression analysis was performed using the JOA recovery rate as the dependent variable (Table 2).

**Table 2:** Logistic regression analysis of “good” and “poor” groups

Variables	*β*	OR	95% CI	*P*
Occupation ratio > 60%	1.301	3.672	1.539–8.762	0.003*
Extension to C2	0.909	2.481	1.039–5.921	0.041*
Open Side (lateral OPLL, ipsi/contra	1.269	3.556	1.308–9.667	0.013*

*There was significant differences between two groups (*P* < 0.05)

*β*, Coefficient estimation; OR, odds ratio; CI, confidence interval

Logistic regression analysis revealed a significant correlation between the occupation ratio (*P* = 0.003), OPLL extending to C2(*P* = 0.041) and JOA recovery rate. Meanwhile, for the patients with laterally deviated OPLL, the difference in the open side was also significantly related to the JOA recovery rate (*P* = 0.013). In addition, an occupation ratio > 60%, OPLL extending to C2 and opening the door on the same side (for lateral OPLL) were independent risk factors affecting the JOA recovery rate (OR > 1).

Patients with OPLL extending to C2 were 2.481-fold more likely to have worse neurological recovery (OR = 2.481) than those without extension. An occupation ratio > 60% was 3.672-fold more likely to be associated with a lower JOA recovery rate (< 50%) than an occupation ratio ≤ 60% (OR = 3.672). The probability of a lower JOA recovery rate (< 50%) in patients with the door open on the ipsilateral side was 3.556-fold that of patients with the door open on the contralateral side among these patients with laterally deviated (OR = 3.556). Except for the axial ossified pattern, the multivariate regression conclusion supported the results of the univariate analysis.

## Discussion

The previous couple of years have witnessed the prevalence of research on OPLL, especially in terms of etiology and therapy. Over the years, laminoplasty has been viewed as a reasonable choice for the treatment of OPLL. However, the follow-up of the patients demonstrated that the postoperative neurological outcomes were not all optimistic, and the factors affecting the postoperative result have always been controversial. The present univariate and multivariate analyses explored the association between the characteristics of ectopic bone and the recovery of neurological function following UODL. Based on our results, patients with an occupying ratio > 60%, OPLL extending to C2 and opening the door on the ipsilateral side (for lateral OPLL) were more likely to exhibit a poor JOA recovery rate. In contrast, age, sex, BMI, average number of ossification levels, type of OPLL, shape of OPLL, K-line state and axial ossified pattern were not identified as significant predictors of functional outcome.

**Fig. 1:**
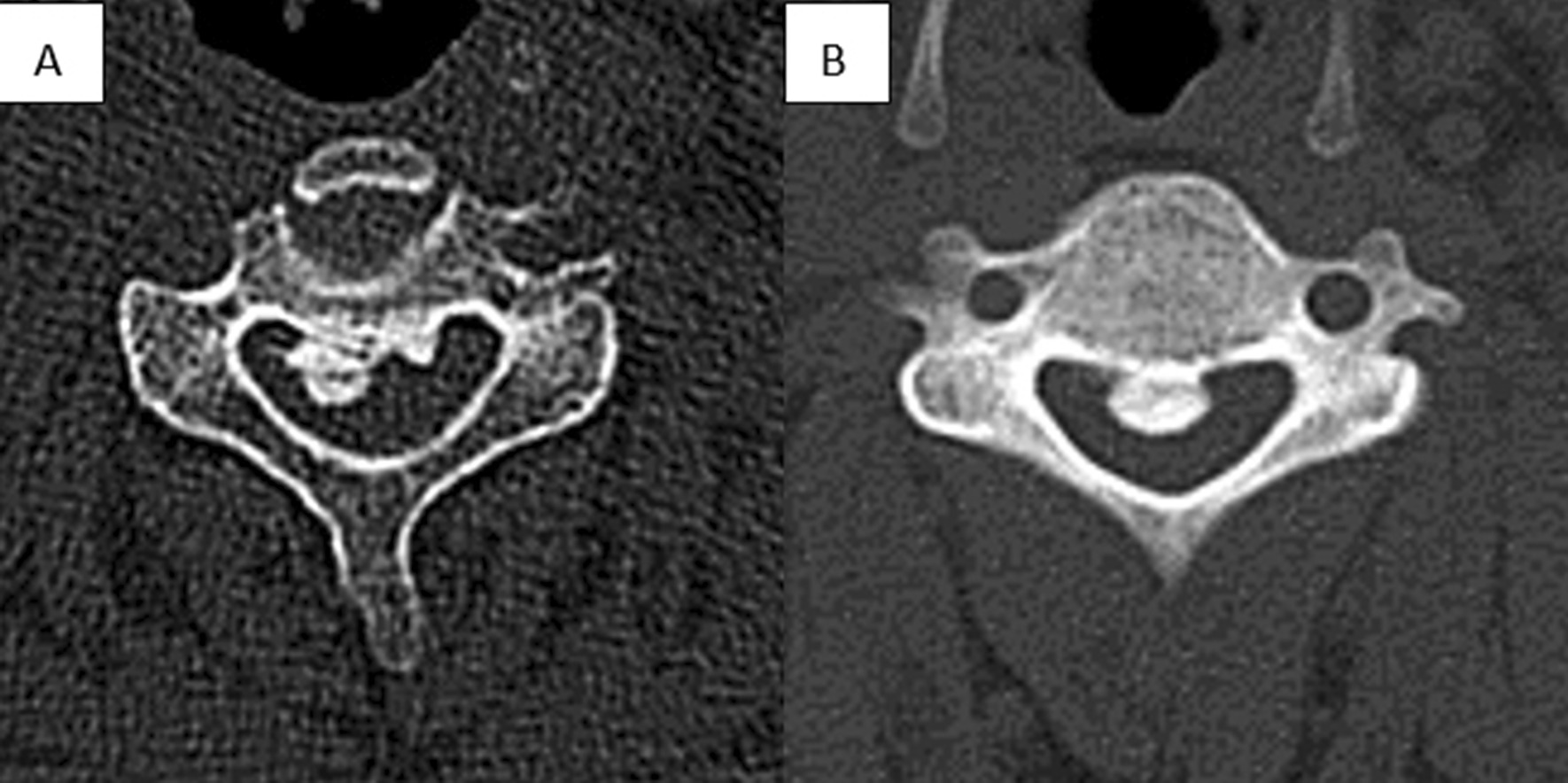
Axial ossified pattern of OPLL on CT. Central OPLL: the posterior prominence of the OPLL is located in the middle one-third of the spinal canal. **A** Lateral OPLL. **B** Central OPLL

Little attention has been given to the axial pattern of ectopic bone. In 2013, Kawaguchi et al. [15] made a new classification of OPLL based on CT images. On axial CT images, it can be divided into the central type and lateral type at the level of the spinal canal with the most obvious ossification. The posterior prominence of the OPLL is located in the middle one-third of the spinal canal, which can be defined as the central type. However, as early as 2008, Matsunaga et al. [16] proposed this OPLL classification and believed that lateral OPLL was a radiographic risk factor for the development of myelopathy. To date, no related studies have revealed the association between axial ossification pattern and neurological outcomes. In our research, we found that the proportion of lateral OPLL in the “poor” group was significantly higher than that in the “good” group, and the difference was statistically significant (*p* = 0.032). This seems to suggest that patients with central ossification can achieve better postoperative results than those with lateral ossification. However, in multivariate analyses, lateral ossification was not a risk factor for postoperative neurological outcomes. At present, we cannot elucidate the exact reason. However, for patients with lateral ossification, we presumed that the different position relationship between the open side and the ectopic bone could affect the recovery of neurological function after the operation. Of course, the contingency caused by a smaller sample size cannot be completely ruled out. Therefore, it is not possible to prove whether lateral ossification has an apparent correlation with poor neurological recovery, and further research is necessary (Fig. 1).

**Fig. 2:**
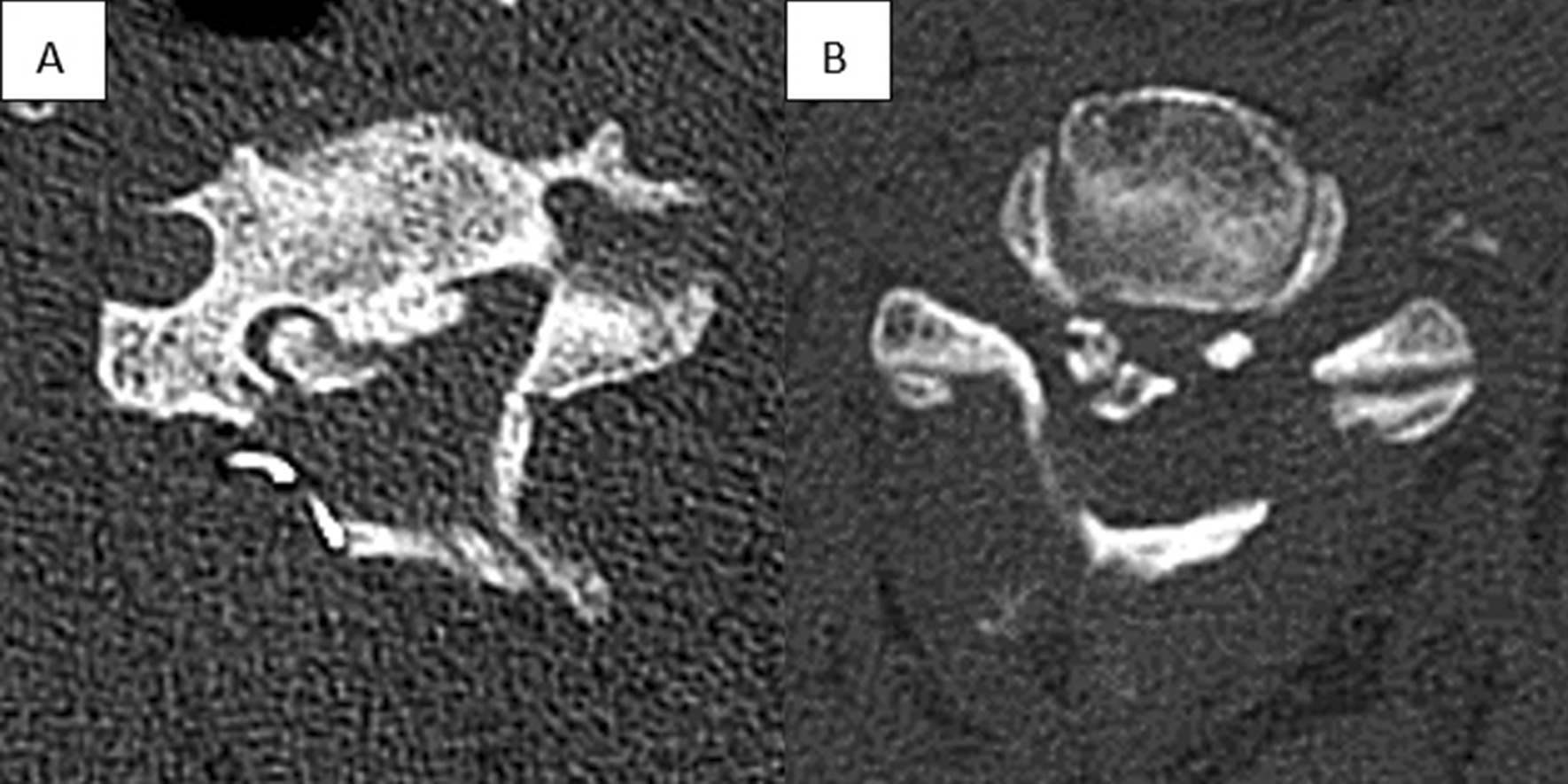
The lateral OPLL can be divided into an open door on the ipsilateral side and an open door on the contralateral side according to the position relationship between the open side and the largest ectopic bone. **A** Open door on the ipsilateral side. **B** Open door on the contralateral side

On this basis, we selected all the patients with lateral ossification, compared the choice of the open side between the “good” group and the “poor” group, and performed logistic regression analysis to explore whether the position relationship between the open side and the ectopic bone affected the outcome of neurological function. Interestingly, when the open side is on the contralateral side of the ectopic bone, it seems that a better recovery of neurological function can be obtained. Coincidentally, Tang et al. [9] came to the same conclusion and believed that using the contralateral lamina as the open side can achieve greater spinal cord area (SCA) enhancement and thus more adequate decompression. However, there was no significant difference in the enlargement rate of the spinal canal in the study, and the exact mechanism has not been elaborated. In our opinion, this may be associated with ischemia–reperfusion of the spinal cord. Opening the door on the ipsilateral side may lead to excessive decompression of the spinal cord located on the severely ossified side, and the probability of ischemia–reperfusion will increase significantly, thus affecting the recovery of postoperative neurological function. While this is only a hypothesis that we have made with the research results, further investigation is worthy (Fig. 2).

The most common classifications of OPLL shape are “plateau” and “hill.” Some scholars revealed that patients with a hill-shaped OPLL had a worse postoperative JOA score than those with plateau-shaped OPLL after laminoplasty [8, 13, 14, 17, 18]. For example, Lwasaki et al. [14] found that the postoperative JOA score was not significantly improved in patients with hilly ossification, and the long-term clinical effect was poor. However, the mechanism is not clear. In our study, the shape of ectopic bone was not a risk factor affecting postoperative neurological recovery. In general, ectopic bone tends to be stable. Although the spinal cord is compressed, the compression is relatively stable. As a result, acute functional deterioration is rare except for injury. In most studies, the shape of ectopic bone (CT image) at the maximum ossification level is generally defined, but the shape of ectopic bone is not completely single. As a result, most of them are mixed, so it is difficult to distinguish the two types completely. In addition, according to our observation, there was no significant difference in the spinal canal enlargement rate of patients after surgery. Therefore, we may speculate that there should be no significant difference in the decompression effect of the spinal cord, namely the extent of expansion and the distance of backward displacement. Nevertheless, further comparative studies are needed to confirm our conjecture.

**Fig. 3:**
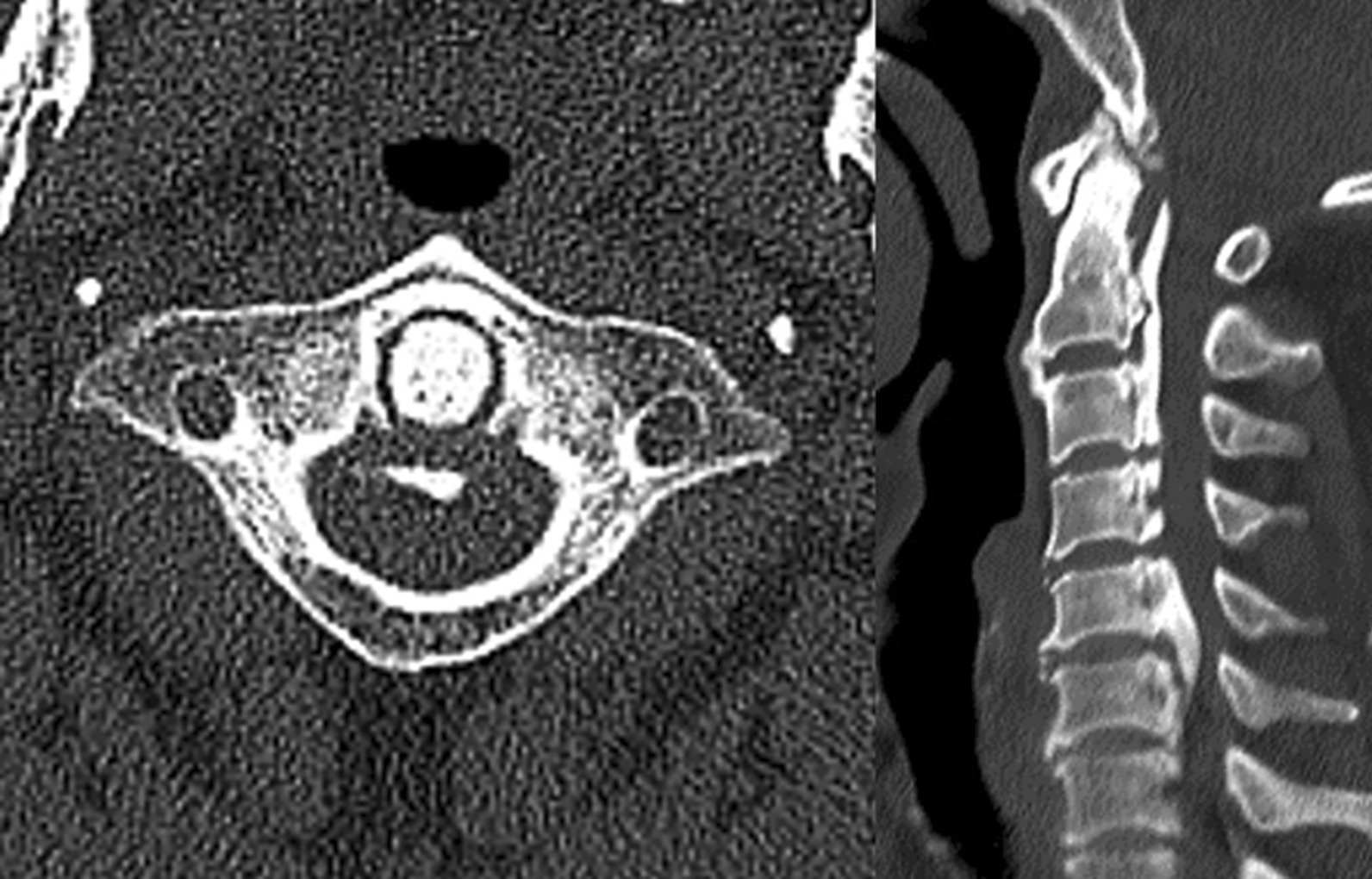
OPLL extending to the upper cervical spine (C1-2)

OPLL extending to C2 and above (upper cervical spine) is rarely mentioned, because the maximum ectopic bone of cervical OPLL is most common in the fourth and fifth segments of the cervical spine, while the incidence of OPLL of the upper cervical spine is relatively low. Consequently, most studies have focused on the lower cervical spine [19]. The correlation between ectopic bone extending to C2 and postoperative neurological outcome is not clear. Lee et al. conducted a study showing that there was no significant difference in surgical outcomes between patients with C2 ossification and patients without C2 ossification [20]. However, this study does not limit the method of operation and the scope of decompression, so the level of evidence is apparently insufficient. In this study, we found that C2 ossification was an independent risk factor for poor outcome after UODL. Although the same conclusion has not been found in previous reports, a study suggested that the JOA recovery rate of patients with C2 ossification was significantly lower than that of patients without C2 ossification; there was no statistical difference [21]. This may be related to different decompression. Based on the current conclusion, we believe that stenosis of the upper cervical spinal canal and an insufficient scope of surgical decompression may be the main reasons for the poor prognosis of patients with ossification of the upper cervical region. In the included samples, the main operative segments were C3-7 and C3-6, and C2 and the above segments were rarely decompressed, which may lead to insufficient relief of spinal cord compression in the upper cervical spine. Of course, the major level of spinal stenosis was not located in the upper cervical spine. Liu et al. [21] also advocated that extended laminoplasty up to the C2 segment could contribute to significant improvements in neurologic function for cases with OPLL located at the C2 segment and needing decompression. However, the structure of the upper cervical vertebra is complex, decompression or not, and decompression methods need to be discussed in a variety of factors. At present, although there are studies in this area [22, 23], they are quite limited and may be used as the focus of studies on cord compression of the upper cervical spine in the future (Fig. 3).

**Fig. 4:**
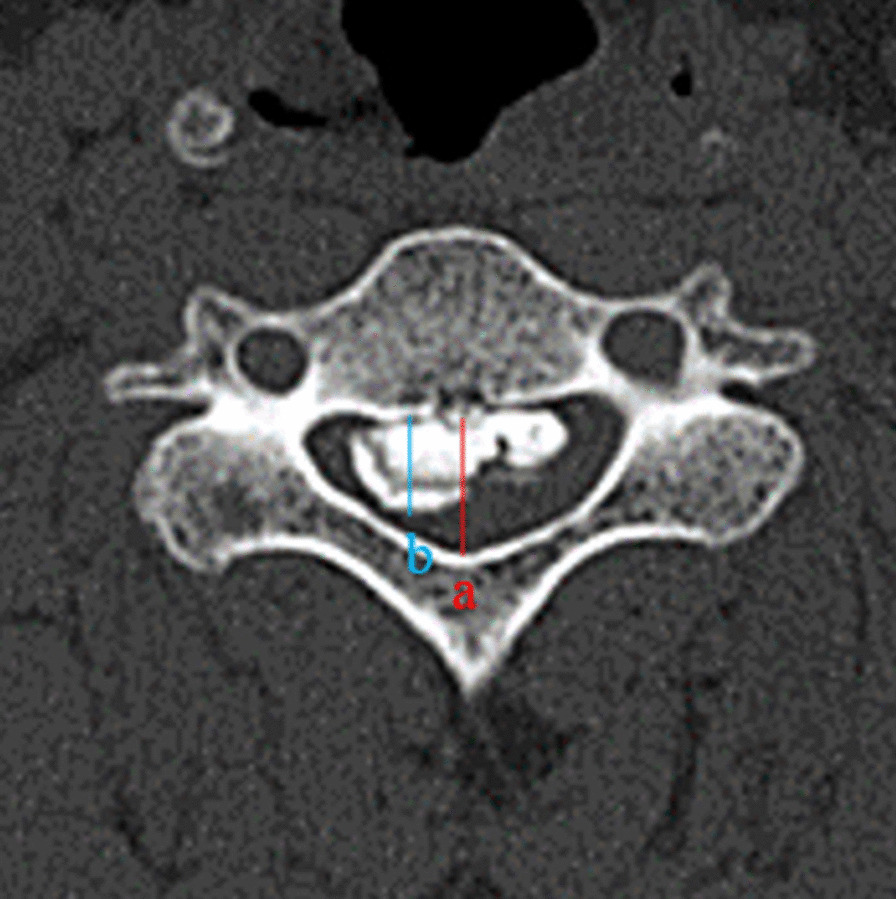
Occupation rate of the spinal canal. **a** Canal diameter; **b** anteroposterior diameter of ossification. Occupation ratio (OR) = *b*/*a* × 100%

The occupying ratio of the spinal canal has a remarkable impact on the prognosis of patients, which has been recognized by most scholars, and it is used as an important reference to determine the plan of operation. Although laminoplasty is regarded as a simple and effective surgical method for the treatment of cervical OPLL [24–28], its effectiveness of in treating patients with anterior massive lesions is doubted. Yamazaki et al. [29] demonstrated a preoperative percent stenosis of more than 50% significantly correlated with postoperative residual cord compression. It is the residue of anterior compression of the spinal cord that will prevent improvements in neurological outcomes. Hirai et al. reported that the surgical outcome of laminoplasty would be poor when the occupying ratio is greater than 50% [30]. At the same time, Chen et al. [31] believed that OPLL patients with a spinal canal occupying ratio greater than 50% were not suitable for laminoplasty. Based on this, we have reason to believe that there is a certain relativity between the occupancy of the spinal canal and the recovery of postoperative neurological function. A larger occupying ratio tends to predict worse neurological function. According to our statistics, an occupying ratio > 60% is an independent risk factor for poor neurological outcome, which is consistent with a previous study by [32]. Fujimori et al. [33] and Iwasaki et al. [14] also advocated that patients with an occupying ratio > 60% had a poor prognosis after laminoplasty (Fig. 4).

Laminoplasty relieves the compression of ectopic bone on the spinal cord by indirect decompression, but for larger ectopic bone, it may be difficult to capture adequate decompression with limited spinal canal enlargement. The limited backward drift of the spinal cord, incomplete expansion of the spinal canal and loss of the postoperative lordosis angle may be the causes of poor neurological recovery. From this point of view, laminoplasty does not seem to be beneficial for patients with a large space occupation of the spinal canal. However, the choice of surgical methods needs to be considered in many aspects, and not all patients with an occupying ratio > 60% have a poor prognosis. Fujimori et al. contended that if cervical lordosis is ≥ 20° preoperatively, even if the occupying rate of OPLL is 60% or more, it is possible to obtain a good prognosis [19].

The idea of the K-line was designed to combine the curvature of cervical vertebra and the size of bone mass into a single parameter, and whether it exceeded the K-line was the choice of surgical approach [11]. He believed that a sufficient posterior shift of the spinal cord and neurologic improvement would not be obtained after posterior decompression surgery in the K-line (−) group. Since the K-line was proposed, most scholars have accepted this idea and regard it as a simple and practical indicator to guide the selection of surgical methods [34]. However, with the development of research, it has been increasingly found that the K-line is not a good predictor of surgical outcome. Takeuchi et al. [35] found that, even for patients demonstrating K-line (+) in the neutral neck position, the K-line (−) in the neck-flexed position affected surgical outcome. On the contrary, Tsujimoto et al. [36] contend that, for patients with K-line (−) OPLL, favorable neurological recoveries could be expected after laminoplasty in cases where the OPLL lesion responsible for cervical myelopathy was in the upper cervical spine or where the K-line changed to (+) in the neck-extended position. Furthermore, it can be hard to clearly see the OPLL and the midpoints of the spinal canal at C7 in the lateral radiograph, and interobserver error sometimes occurs. This demonstrates that the traditional K-line cannot completely determine the effect of the operation.

At present, this study has some limitations: 1. The current study is a small-scale retrospective data analysis of a single institution using a small sample size, and the value of the statistical results is limited. Meanwhile, this is a short-term postoperative study, and it is not possible to evaluate the long-term prognosis. Therefore, future large-scale prospective multicenter studies are needed. 2. The included parameters are limited. We mainly analyzed some characteristics of ectopic bone that may be associated with postoperative neurological outcomes, but did not include comprehensive OPLL variables, which may lead to the omission of some risk factors. Nevertheless, increasing the number of elements to be examined could complicate the analysis, and so we could not include all factors in our article. In this study, only the JOA recovery rate was used as the criterion to evaluate the postoperative neurological outcome, but sometimes the recovery rate could not fully reflect the recovery of neurological function. The combination of multiple evaluation indices of neurological function may be a better evaluation standard.

## Conclusion

The study analyzes the risk factors affecting postoperative neurological outcomes. In our study, an occupation ratio > 60% is the most important risk factor. OPLL extending to C2 is another one. Therefore, the design of the surgical plan should comprehensively consider the extent and scope of decompression. Meanwhile, opening the door on the ipsilateral side is indeed a risk factor for lateral OPLL. Better neurological function may be obtained by choosing the opposite side of the heterotopic bone as the open side.

## Data Availability

All the data will be available upon motivated request to the corresponding author of the present paper.

## Abbreviations

JOA: Japanese Orthopedic Association
OPLL: Ossification of posterior longitudinal ligament
OR: Occupation rate
UODL: Unilateral open-door laminoplasty
BMI: Body mass index
